# Supplementary material for: The Immune Efficacy of Inactivated Pseudorabies Vaccine Prepared from FJ-2012ΔgE/gI Strain
Source: Microorganisms. 2022 Sep 21;10(10):1880. doi: 10.3390/microorganisms10101880 (PMC9612264; doi:10.3390/microorganisms10101880)
Supplement: Supplementary file 1 [file microorganisms-10-01880-s001.zip › Table S1. The results of piglets PRV gB and gE antibody via serological ELISA before vaccination..pdf]

Table S1. The results of piglets PRV gB and gE antibody via serological ELISA before vaccination.

Serum sample No.	gB antibody [a]		gE antibody [b]	
	S/N	Result	S/N	Result
FJ-2012-GEL*-1	1.17	Negative	1.00	Negative
FJ-2012-GEL* 2	1.27	Negative	1.01	Negative
FJ-2012-GEL*-3	1.24	Negative	0.97	Negative
FJ-2012-GEL*-4	1.33	Negative	1.10	Negative
FJ-2012-GEL*-5	1.16	Negative	1.08	Negative
FJ-2012-VG*-1	1.17	Negative	1.15	Negative
FJ-2012-VG*-2	1.20	Negative	1.03	Negative
FJ-2012-VG*-3	1.05	Negative	1.11	Negative
FJ-2012-VG*-4	1.09	Negative	1.05	Negative
FJ-2012-VG*-5	1.19	Negative	1.06	Negative
vPRV*-1	1.23	Negative	0.93	Negative
vPRV*-2	1.20	Negative	0.99	Negative
vPRV*-3	1.11	Negative	0.99	Negative
vPRV*-4	1.20	Negative	0.94	Negative
vPRV*-5	1.27	Negative	1.05	Negative
K61-1	1.24	Negative	1.11	Negative
K61-2	1.15	Negative	0.98	Negative
K61-3	1.12	Negative	0.98	Negative
K61-4	1.03	Negative	0.99	Negative
K61-5	1.08	Negative	0.99	Negative
Unimmunized challenged-1	1.12	Negative	1.04	Negative
Unimmunized challenged-2	1.17	Negative	0.96	Negative
Unimmunized challenged-3	1.20	Negative	1.06	Negative
Unimmunized challenged-4	1.20	Negative	1.03	Negative
Unimmunized challenged-5	1.19	Negative	1.00	Negative
Control-1	1.03	Negative	0.97	Negative
Control-2	1.14	Negative	1.04	Negative
Control-3	1.06	Negative	1.04	Negative
Control-4	0.99	Negative	1.02	Negative
Control-5	0.90	Negative	1.04	Negative

[a] gB-specific antibody levels: a sample with S/N ratios $\geqslant$ 0.70 were classified as negative for gB antibodies; [b] gE-specific antibody levels: a sample with S/N ratios $\geqslant$ 0.70 were classified as negative for gE antibodies. The "*" mark in the group top right indicates an inactivated vaccine.
