# Supplementary figures and images for: The Immune Efficacy of Inactivated Pseudorabies Vaccine Prepared from FJ-2012ΔgE/gI Strain
Source: Microorganisms. 2022 Sep 21;10(10):1880. doi: 10.3390/microorganisms10101880 (PMC9612264; doi:10.3390/microorganisms10101880)

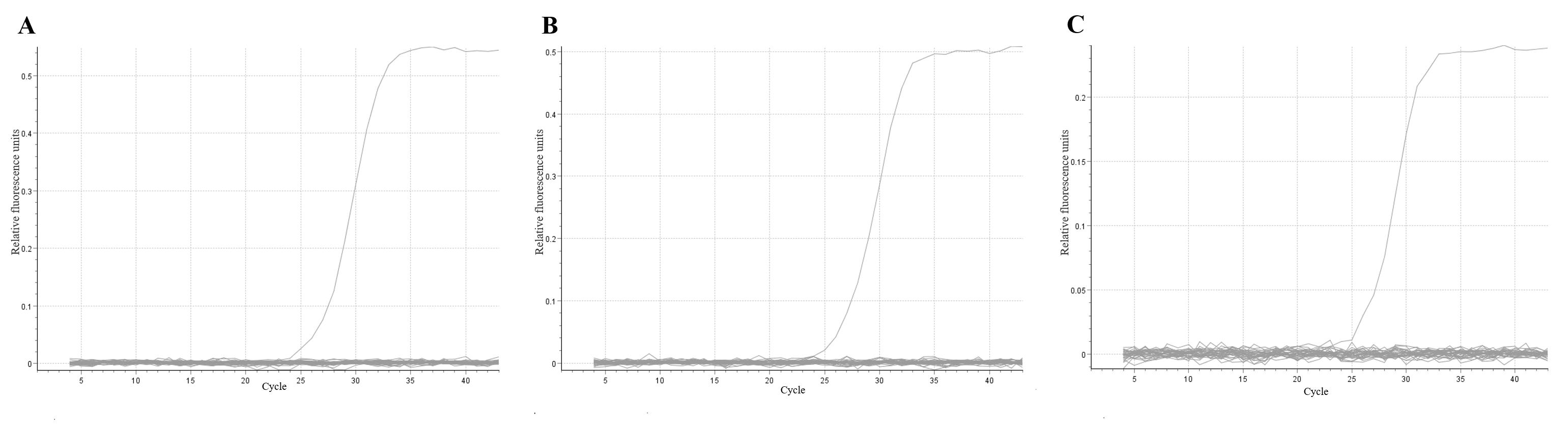

Supplement: Supplementary file 1 [file microorganisms-10-01880-s001.zip › Figure S1. The gum swab test results of CSFV(A), PRRSV(B), and PRV(C) by RT-PCR in the experimental piglets before vaccination..tif]
